# A High-Sensitivity MEMS Pressure Sensor with a Narrow Cross-Beam Membrane–Short Beam Structure

**DOI:** 10.3390/mi17091062

**Published:** 2026-09-08

**Authors:** Tao Wang, Zibang Xiao, Peicang Chen, Meng Nie, Zhen Yan, Shijie Deng, Jing Chen, Xiaolin Wang, Jingquan Liu

**Affiliations:** 1 National Key Laboratory of Advanced Micro and Nano Manufacture Technology, Shanghai Jiao Tong University, Shanghai 200240, China; viola9876543210@163.com (J.C.); xlwang83@sjtu.edu.cn (X.W.); jqliu@sjtu.edu.cn (J.L.); 2 Wuxi Zhongwei Microchips Co., Ltd., Wuxi 210093, China; chenpc262024@163.com; 3 Key Laboratory of MEMS of the Ministry of Education, School of Integrated Circuits, Southeast University, Nanjing 210096, China; 15107960067@163.com (Z.X.); 18863680592@163.com (Z.Y.); dengshijie0225@163.com (S.D.); 4 School of Mechano-Electronic Engineering, Xidian University, Xi’an 710071, China

**Keywords:** microelectromechanical systems, pressure sensor, finite-element method, sensitivity, nonlinear optimization

## Abstract

To address the difficulty of balancing sensitivity and nonlinearity in MEMS piezoresistive pressure sensors, this study proposes an MEMS piezoresistive pressure sensor with a narrow cross-beam membrane–short beam structure. The structure introduces a tapered design at the ends of a conventional cross beam and incorporates short beams to enhance stress concentration in the sensitive regions, thereby improving output sensitivity while maintaining low nonlinearity. Finite-element analysis was performed to evaluate the stress distribution and deflection characteristics and to compare the proposed structure with conventional cross-beam and other diaphragm structures. Under identical overall dimensions, the proposed structure improves sensitivity by 61% relative to the conventional cross beam. Based on the finite-element results, multivariate fitting models for surface stress and deflection were established, and nonlinear optimization was used to determine the constrained optimal geometrical parameters within the validated design domain. Simulation results indicate that, over a pressure range of 0–1 kPa, the proposed sensor achieves a sensitivity of 12.23 mV/V/kPa and a maximum nonlinearity of 0.196% FSS, demonstrating favorable performance for micropressure detection.

## 1. Introduction

A microelectromechanical system (MEMS) is a miniaturized system that integrates micromechanical structures, electrical units, and signal-processing components using micro-/nanofabrication and integrated-circuit technologies. MEMS pressure sensors are among the most widely used MEMS sensors [1] and were also among the earliest MEMS devices to be developed and adopted across numerous industries. A MEMS pressure sensor uses a micromechanical sensing element to detect an external pressure signal, after which a conversion circuit transforms the pressure signal into a voltage signal that can be processed and recognized by the system, enabling the actuator to perform the corresponding operation. According to their operating principles, MEMS pressure sensors can be classified as piezoelectric, capacitive, fiber-optic, resonant, and piezoresistive pressure sensors [2]. Among these sensing mechanisms, piezoresistive pressure sensors offer the advantages of direct electrical readout, a relatively simple signal-conditioning circuit based on the Wheatstone bridge, and compatibility with mature silicon micromachining and integrated-circuit fabrication processes. In addition, their capability for static and quasi-static pressure measurement makes them particularly suitable for micropressure sensing applications [3].

Existing diaphragm-engineering strategies can be organized according to the mechanism used to redistribute stress. Beam-reinforced diaphragms introduce local stiffness discontinuities that guide stress toward the beam roots and diaphragm edges. Tian et al. [4] incorporated cross beams into a planar square diaphragm, while Li et al. [5,6] subsequently investigated a four-beam configuration through numerical optimization and experimental fabrication. These relatively regular layouts are compatible with conventional micromachining; however, the results summarized in Table 1 indicate that beam reinforcement alone provides only a moderate improvement in sensitivity. These results indicate that further optimization of the beam geometry, particularly the beam width and beam-end transition, may provide additional opportunities for enhancing stress concentration.

Local stiffening using islands, bosses, or peninsula features constitutes a second route. Single-island [7], double-island [8], four-island [9], peninsula [10], and central-island–four-narrow-beam [11] structures alter the diaphragm deformation pattern and transfer stress toward selected piezoresistive regions. Several island-based designs achieve high sensitivity, but their nonlinearities differ markedly; the single-island and double-island structures listed in Table 1 exhibit relatively large nonlinearities, whereas the four-island structure achieves a more favorable balance.

Rather than locally stiffening the diaphragm, groove-based and locally thinned structures enhance the mechanical response by reducing stiffness in selected regions. The groove–cross-beam design [12] and the locally thinned diaphragm [13] exemplify this approach. Although local compliance can amplify deformation and stress in the sensitive regions, the resulting performance becomes more dependent on groove depth or local membrane thickness and is therefore more sensitive to dimensional variations introduced during etching.

Composite approaches combine two or more stress-redistribution mechanisms. Representative examples include a cross-beam–peninsula diaphragm [14], a four-petal diaphragm incorporating short narrow beams and a central boss [15], and a composite structure comprising a central circular island, cross beams, four peninsula features, and short narrow beams [16]. These designs provide additional control over the stiffness distribution and can achieve a favorable balance between sensitivity and nonlinearity. Nevertheless, the increased number of interacting structural features also increases the number of design variables and the complexity of optimization and fabrication.

The operating ranges, sensitivities, and nonlinearities of these representative MEMS piezoresistive pressure sensors are summarized in Table 1 for comparison.

Taken together, previous studies reveal a recurring trade-off among stress concentration, output linearity, and structural complexity. Regular beam-reinforced diaphragms offer structural simplicity but limited stress concentration; island, peninsula, groove, and locally thinned diaphragms strengthen the local response but may increase nonlinearity or sensitivity to critical dimensions; and highly composite structures provide greater control over stress redistribution at the expense of geometrical and process complexity [17,18]. Therefore, improving sensitivity and linearity without introducing excessive structural complexity remains an important challenge in the design of MEMS piezoresistive pressure sensors. To address this challenge, this study proposes an MEMS piezoresistive pressure sensor with a narrow cross-beam membrane–short-beam structure. The cross-beam ends are tapered to strengthen stress transfer toward the piezoresistive regions, and short-beam segments are introduced to tailor the local stiffness and deformation. Finite-element analysis is used to characterize the stress and deflection distributions, after which multivariate fitting and constrained nonlinear optimization are applied to determine the structural dimensions. The proposed design is intended to improve stress concentration and output linearity while retaining a relatively regular geometry compatible with conventional bulk-silicon micromachining.

## 2. Operating Principle

### 2.1. Piezoresistive Effect and Piezoresistive Coefficients

The piezoresistive effect describes the change in semiconductor resistivity caused by mechanical stress [19]. For a resistor located on the diaphragm, the resistance variation is governed primarily by the longitudinal stress $\sigma_{l}$ and transverse stress $\sigma_{t}$ [20]. Owing to the anisotropic piezoresistive properties of single-crystal silicon [21], P-type silicon piezoresistors are commonly aligned along the <110> direction to obtain a large piezoresistive response [19,20]. For P-type silicon in this orientation, the comparatively small $\pi_{11}$ and $\pi_{12}$ coefficients can be neglected. Thus, the longitudinal and transverse piezoresistive coefficients can be approximated as $\pi_{l}\approx \pi_{44}⁄2$ and $\pi_{t}\approx -\pi_{44}⁄2$, respectively. Accordingly, the relative resistance change can be expressed as follows [22]:

(1) $$\frac{∆R}{R}=\pi_{l}\sigma_{l}+\pi_{t}\sigma_{t}=\frac{\pi_{44}}{2}\left(\sigma_{l}-\sigma_{t}\right)=\frac{\pi_{44}}{2}\Delta \sigma$$

Here, $\sigma_{l}$ and $\sigma_{t}$ denote the longitudinal and transverse stresses acting on the piezoresistor, respectively. The longitudinal–transverse stress difference is defined as $\Delta \sigma =\sigma_{l}-\sigma_{t}$. According to Equation (1), the relative resistance change of a piezoresistor is related to the longitudinal–transverse stress difference in the region where it is located, with the stress evaluated as the surface-averaged value over the piezoresistor area.

### 2.2. Wheatstone Bridge

**Figure 1:**
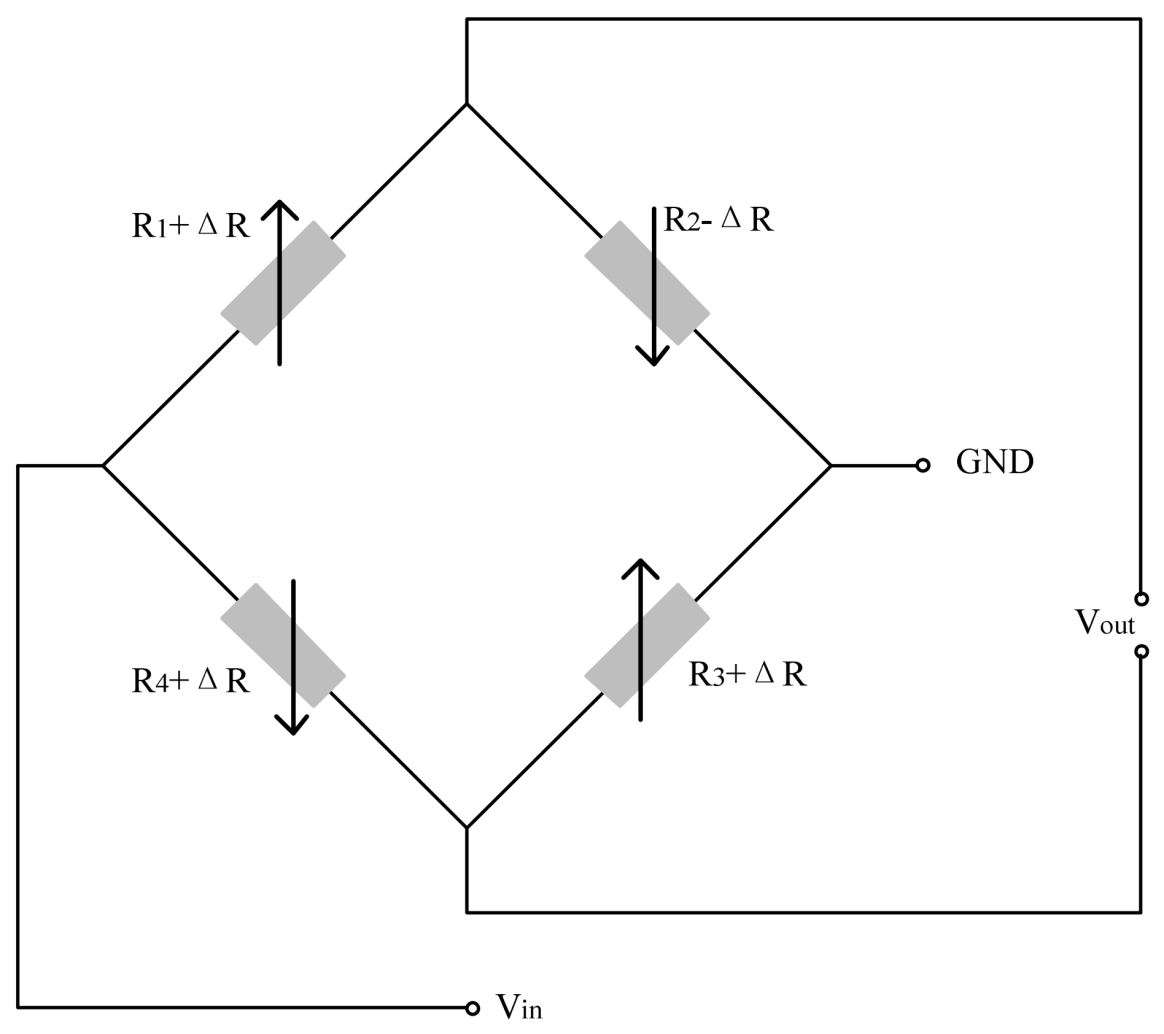
Wheatstone bridge circuit. The arrows indicate the directions of resistance change under applied pressure.

The Wheatstone bridge [23] is shown in Figure 1. When a uniformly distributed pressure is applied normal to the sensor diaphragm, the diaphragm deforms, generating longitudinal and transverse stresses in the regions occupied by the four piezoresistors *R*1, *R*2, *R*3, and *R*4. Owing to the symmetry of the sensor structure and piezoresistor layout, *R*1 and *R*3 form one symmetric pair, whereas *R*2 and *R*4 form the other; therefore, the two piezoresistors within each pair experience approximately identical stress states and resistance changes. The magnitudes of the resistance changes of the *R*1/*R*3 and *R*2/*R*4 pairs are denoted by $\left|{\Delta R}_{1,3}\right|$ and $\left|{\Delta R}_{2,4}\right|$, respectively. Under the applied pressure, the longitudinal–transverse stress differences in the two pairs have opposite signs; consequently, the resistances of *R*1 and *R*3 increase, whereas those of *R*2 and *R*4 decrease [24]. If all four resistors have the same initial resistance R, their loaded resistances satisfy $R_{1}^{\prime }=R_{3}^{\prime }=R+\left|{\Delta R}_{1,3}\right|$ and $R_{2}^{\prime }=R_{4}^{\prime }=R-\left|{\Delta R}_{2,4}\right|$. Based on the voltage–divider relationships of the four resistors [25], the bridge output voltage is

(2) $$V_{out}=(\frac{R_{3}^{\prime }}{R_{3}^{\prime }+R_{4}^{\prime }}-\frac{R_{2}^{\prime }}{R_{1}^{\prime }+R_{2}^{\prime }})V_{in}\;=\left(\frac{R+\left|\Delta R_{1,3}\right|}{\left(R+\left|\Delta R_{1,3}\right|\right)+\left(R-\left|\Delta R_{2,4}\right|\right)}-\frac{R-\left|\Delta R_{2,4}\right|}{\left(R+\left|\Delta R_{1,3}\right|\right)+\left(R-\left|\Delta R_{2,4}\right|\right)}\right)V_{in}\;=\frac{\left|\Delta R_{1,3}\right|+\left|\Delta R_{2,4}\right|}{2R+(\left|\Delta R_{1,3}\right|-\left|\Delta R_{2,4}\right|)}V_{in}$$

Because the sensor structure and the arrangement of the four piezoresistors are symmetric, the magnitudes of the resistance changes of the two pairs differ only slightly under a uniformly distributed normal pressure. Thus, $\left|{\Delta R}_{1,3}\right|-\left|{\Delta R}_{2,4}\right|$ is negligible compared with 2R, and Equation (2) can be simplified as

(3) $$V_{out}=\frac{\left|\Delta R_{1,3}\right|+\left|\Delta R_{2,4}\right|}{2R}V_{in}\;=\frac{1}{2}(\frac{\left|\Delta R_{1,3}\right|}{R}+\frac{\left|\Delta R_{2,4}\right|}{R})V_{in}$$

According to Equation (1), the relative resistance changes of the two piezoresistor pairs are related to their respective longitudinal–transverse stress differences as follows:

(4) $$\frac{\left|\Delta R_{1,3}\right|}{R}=\frac{\pi_{44}}{2}\left|∆\sigma_{1,3}\right|$$

(5) $$\frac{\left|\Delta R_{2,4}\right|}{R}=\frac{\pi_{44}}{2}\left|∆\sigma_{2,4}\right|$$

Substitution of Equations (4) and (5) into Equation (3) yields

(6) $$V_{out}=\frac{1}{2}\times \frac{\pi_{44}}{2}(\left|∆\sigma_{1,3}\right|+\left|∆\sigma_{2,4}\right|)V_{in}$$

Because the longitudinal–transverse stress differences in the *R*1/*R*3 and *R*2/*R*4 regions have opposite signs, $\left|{\Delta \sigma }_{1,3}\right|+\left|{\Delta \sigma }_{2,4}\right|=\left|{\Delta \sigma }_{1,3}-{\Delta \sigma }_{2,4}\right|$. Equation (6) can therefore be written as

(7) $$V_{out}=\frac{\pi_{44}}{4}(\left|∆\sigma_{1,3}-∆\sigma_{2,4}\right|)V_{in}$$

Here, ${\Delta \sigma }_{1,3}$ and ${\Delta \sigma }_{2,4}$ denote the longitudinal–transverse stress differences in the piezoresistive regions of *R*1/*R*3 and *R*2/*R*4, respectively. The bridge stress difference is defined as ${\Delta \sigma }_{bridge}=\left|{\Delta \sigma }_{1,3}-{\Delta \sigma }_{2,4}\right|$. This parameter characterizes the differential stress response between the two piezoresistor pairs and can be used to evaluate the potential sensitivity of the sensor; it is not itself the sensitivity.

## 3. Structural Design and Performance Evaluation

### 3.1. Structural Design

For an MEMS piezoresistive micropressure sensor, the diaphragm structure strongly affects the stress distribution, deflection, and output sensitivity under applied pressure [26]. A conventional square flat diaphragm is simple and readily fabricated; however, its limited stress concentration under micropressure produces only small stress variations in the piezoresistive regions, making high output sensitivity difficult to achieve. A cross-beam structure introduces local stiffness variations into the diaphragm and directs stress toward the beam roots and edges, thereby increasing the stress response at the piezoresistors. Nevertheless, the degree of stress concentration remains constrained by the beam-end transition geometry and beam width, leaving scope for further sensitivity enhancement.

**Figure 2:**
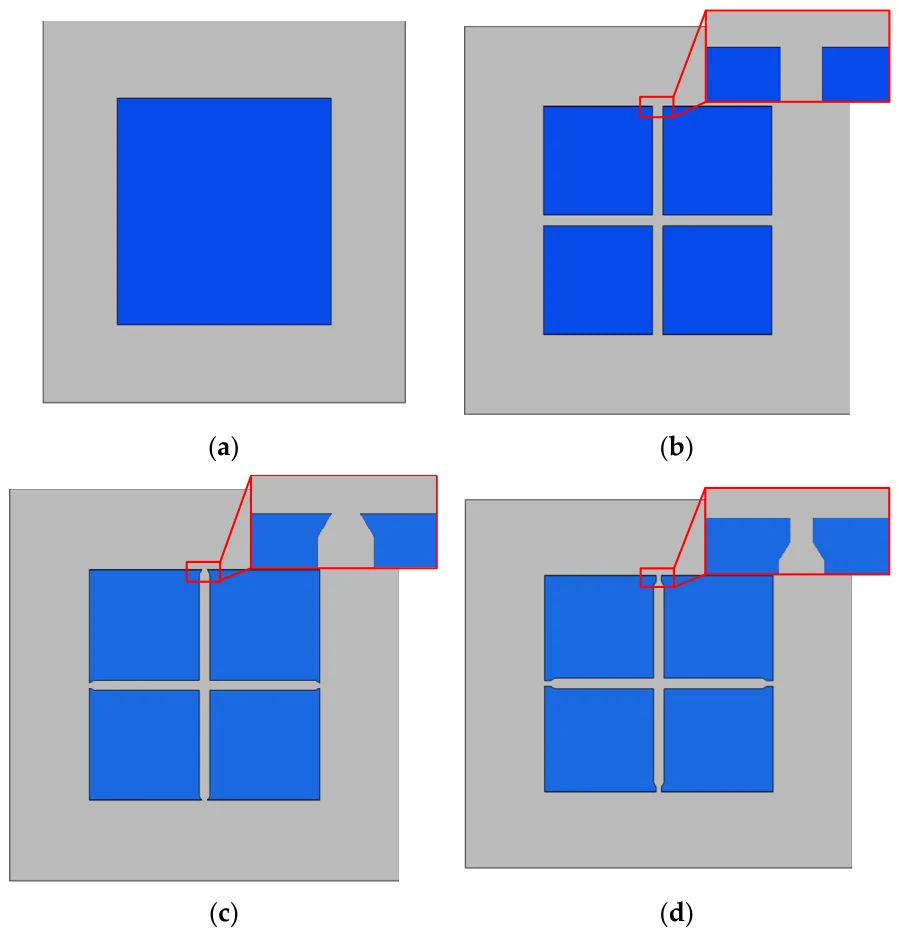
Schematics of the four structural configurations. (**a**) Flat; (**b**) CBM; (**c**) NCBM; (**d**) NCBM-SB.

Based on the preceding analysis, a progressive structural design strategy was adopted. Tapered and short-beam features were introduced successively into conventional flat and cross-beam diaphragms. The tapered feature modifies the stress-transfer path at the ends of the cross beams and further concentrates stress in the sensitive regions, whereas the short beams locally reduce structural stiffness and enhance local deformation under pressure. To systematically evaluate the effects of these structural modifications, four diaphragm configurations were established for comparative analysis, as shown in Figure 2.

Case 1 is the baseline configuration and employs a conventional square flat diaphragm (Flat), as shown in Figure 2a.

Case 2 employs a conventional cross-beam membrane (CBM), as shown in Figure 2b.

Case 3 applies end tapering to the cross beams in Case 2, forming a narrow cross-beam membrane (NCBM), as shown in Figure 2c.

**Figure 3:**
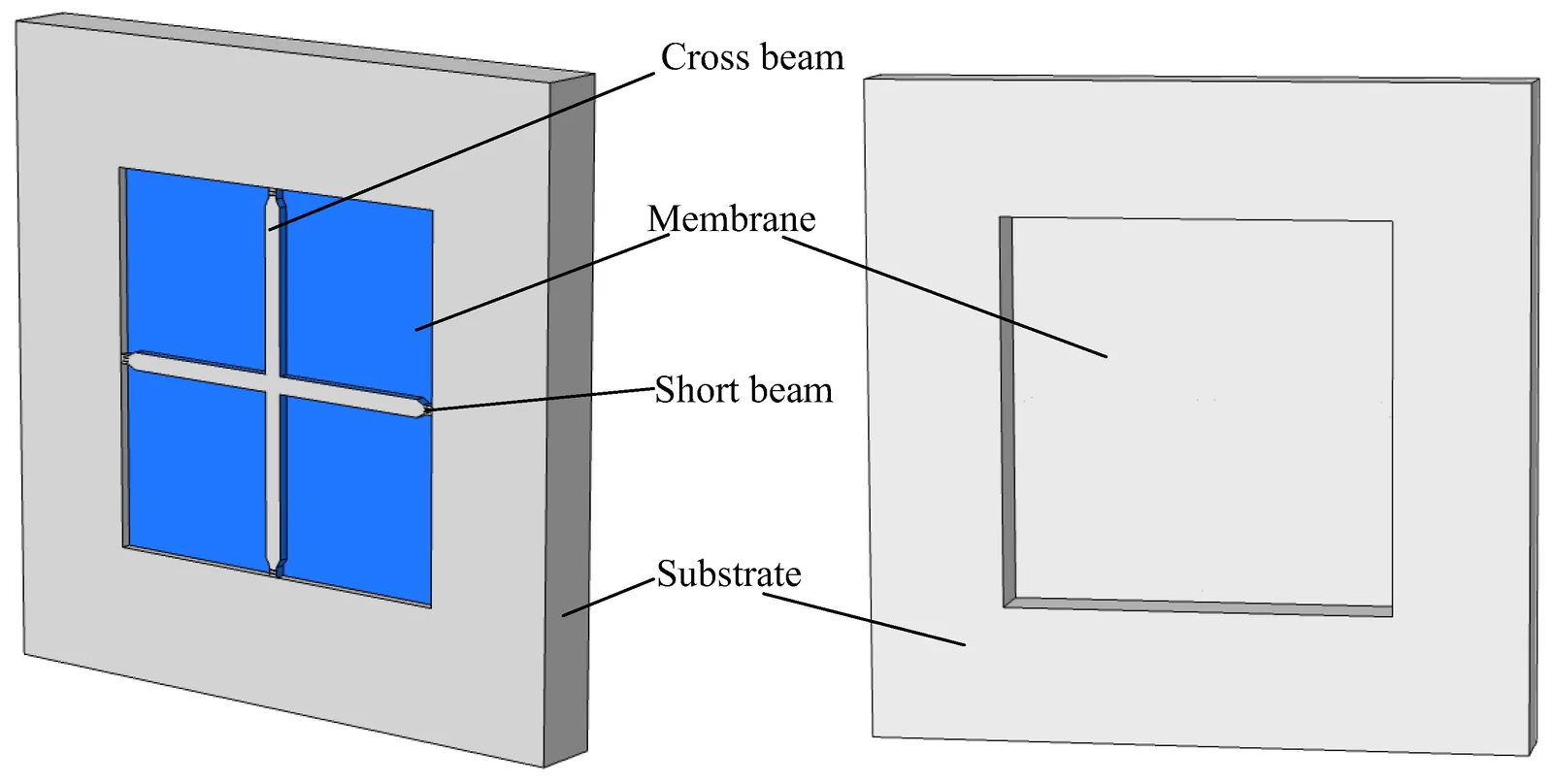
Front- and back-side schematic views of the proposed NCBM-SB sensor. The left view shows the front-side membrane, cross beams, short beams, and substrate; the right view shows the back-side membrane and surrounding substrate.

**Figure 4:**
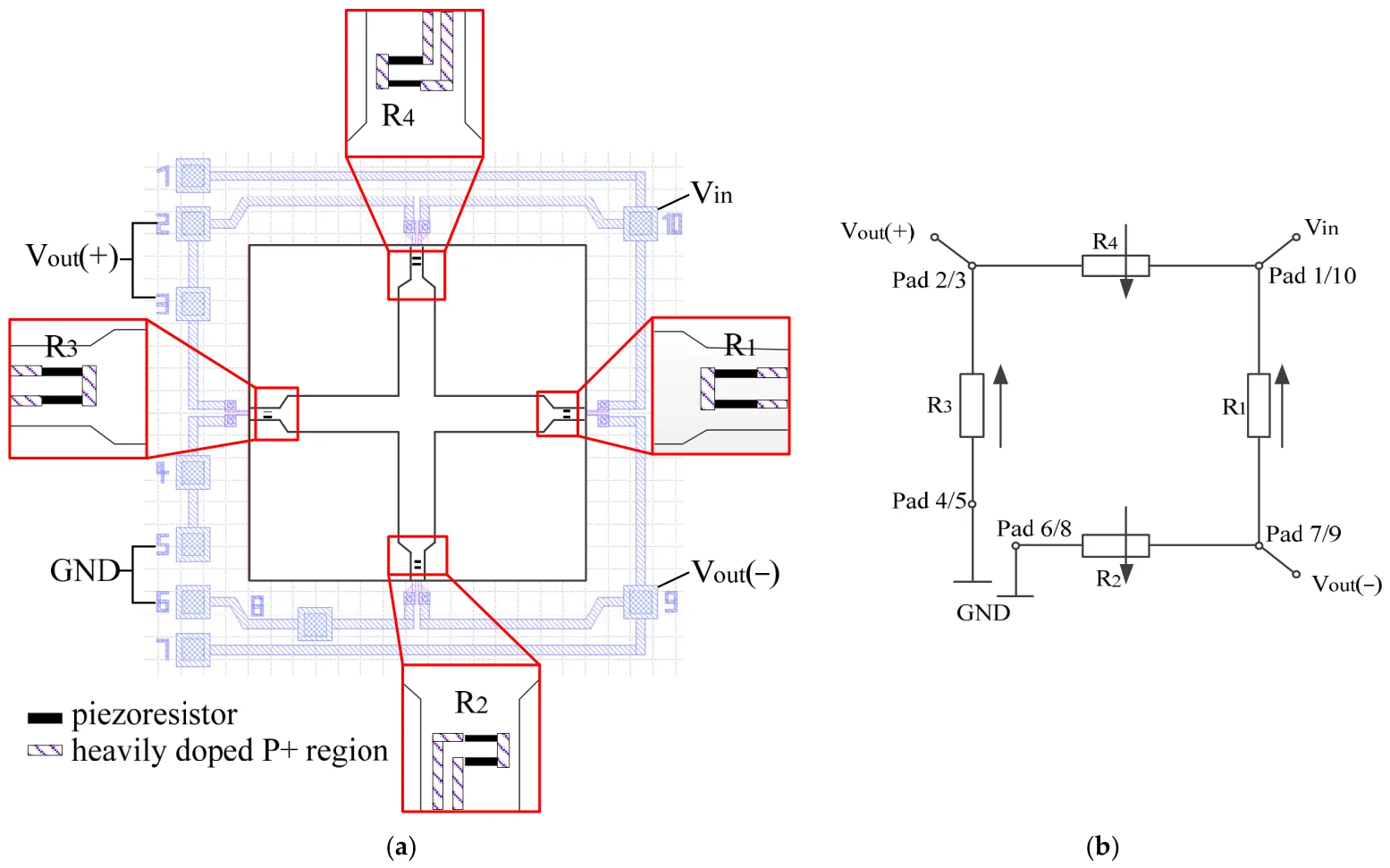
Physical layout and corresponding Wheatstone bridge connection of the proposed pressure sensor. (**a**) Physical layout of the sensor, where the grid area represents the electrical interconnection layout on the sensor surface; (**b**) corresponding Wheatstone bridge configuration, where the arrows indicate the increase or decrease in the resistance of each piezoresistor under applied pressure.

Case 4 is the final structural configuration. Based on Case 3, each cross beam is first tapered and then extended by a short-beam segment, forming a narrow cross-beam membrane with short beams (NCBM-SB), as shown in Figure 2d. The red-boxed insets in Figure 2b–d provide enlarged views of the beam-end transition regions, highlighting the conventional, tapered, and tapered-with-short-beam geometries, respectively. Among these four configurations, Case 4 incorporates both the tapered cross-beam ends and short-beam segments and represents the proposed NCBM-SB sensor structure investigated in this study. The detailed front- and back-side structural views of the proposed NCBM-SB sensor are shown in Figure 3. Figure 4 shows the physical layout of the proposed pressure sensor and the corresponding Wheatstone bridge connection. In Figure 4a, the numbered regions 1–10 denote the electrical pads, which are provided for subsequent electrical characterization of the sensor. The piezoresistors are interconnected through heavily doped P+ regions to form the electrical routing on the sensor surface. Figure 4b shows the Wheatstone bridge configuration corresponding to the physical layout in Figure 4a, illustrating how the four piezoresistors are electrically connected to form the bridge.

In accordance with the actual fabrication process, the beam structure is successively covered by a 450 nm thick SiO_2_ layer and a 425 nm thick Si_3_N_4_ layer. The SiO_2_ layer provides electrical insulation between the silicon sensing structure and the surface interconnects while passivating the silicon surface, whereas the Si_3_N_4_ overlayer provides additional surface passivation and protects the underlying piezoresistors and interconnects from moisture and process-related contamination. The complementary insulation and protective properties of the two dielectric materials account for their combined use in the device. The nominal thicknesses were adopted directly from the actual fabrication process to provide continuous dielectric coverage and the required insulation and passivation performance while remaining compatible with the subsequent patterning steps.

The four configurations have identical principal diaphragm dimensions: a diaphragm size of 1300 μm × 1300 μm, a diaphragm thickness of 6 μm, a beam width of 60 μm, and a beam height of 10 μm. The primary objective is to compare the four diaphragm designs and maximize output sensitivity while ensuring that the maximum central displacement does not exceed one-fifth of the diaphragm thickness, thereby satisfying the applicability condition of small-deflection theory. All analyses were conducted over a pressure range of 0–1 kPa.

### 3.2. Performance Evaluation Method

Finite-element analyses of the four diaphragm structures were performed using COMSOL Multiphysics 5.4. The mechanical and electrical performances of the four configurations were evaluated in terms of maximum central deflection ω, bridge stress difference ${\Delta \sigma }_{bridge}$, bridge output voltage, sensitivity, and nonlinearity. Accordingly, the 450 nm SiO_2_ layer and the 425 nm Si_3_N_4_ layer were explicitly retained in the finite-element model rather than treating the beams as bare silicon structures. All stress and deflection data used in the subsequent parameter fitting and dimensional optimization were obtained from this complete Si/SiO_2_/Si_3_N_4_ multilayer model.

The sensitivity S is defined as the output-voltage variation per unit pressure normalized by the supply voltage and is calculated using the following expression:

(8) $$S=\frac{V_{out}\left(P_{FS}\right)-V_{out}(0)}{V_{in}P_{FS}}$$

In this expression, $V_{out}\left(P_{FS}\right)$ and $V_{out}\left(0\right)$ are the output voltages at the full-scale pressure $P_{FS}$ and zero pressure, respectively, and $V_{in}$ is the bridge supply voltage.

The nonlinearity was evaluated using the endpoint method. The reference output at pressure $P_{i}$ is calculated using the following expression:

(9) $$V_{L}\left(P_{i}\right)=V_{out}\left(0\right)+\frac{V_{out}\left(P_{FS}\right)-V_{out}\left(0\right)}{P_{FS}}P_{i}$$

In this expression, $V_{L}\left(P_{i}\right)$ represents the output of the endpoint reference line at pressure $P_{i}$.

The full-scale span (FSS) is defined as the absolute difference between the output voltages at full-scale pressure and zero pressure:

(10) $$FSS=\left|V_{out}\left(P_{FS}\right)-V_{out}\left(0\right)\right|$$

The nonlinearity is defined as the maximum absolute deviation of the simulated output from the endpoint reference line normalized by the FSS. It is calculated using the following expression:

(11) $$\delta_{NL}=\frac{max\left|V_{out}\left(P_{i}\right)-V_{L}\left(P_{i}\right)\right|}{FSS}\times 100\%$$

Therefore, the calculated nonlinearity is reported in % FSS, representing the maximum output deviation as a percentage of the full-scale span.

### 3.3. Comparison of the Four Structural Configurations

**Figure 5:**
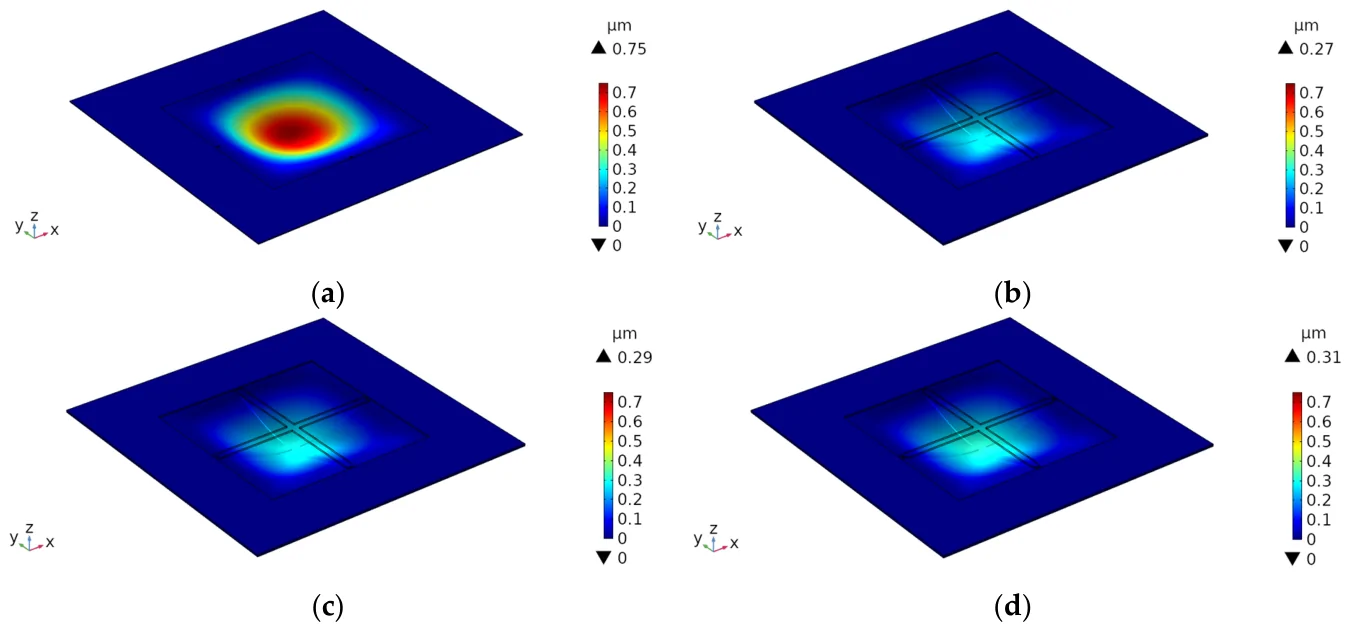
Deflection distributions of the four configurations under a pressure load of 1 kPa. (**a**) Flat; (**b**) CBM; (**c**) NCBM; (**d**) NCBM-SB.

The central deflections ω of the four configurations under a pressure load of 1 kPa are shown in Figure 5. Case 1 exhibits the largest central deflection, whereas the deflections of Cases 4, 3, and 2 are similar and substantially smaller than that of Case 1.

**Figure 6:**
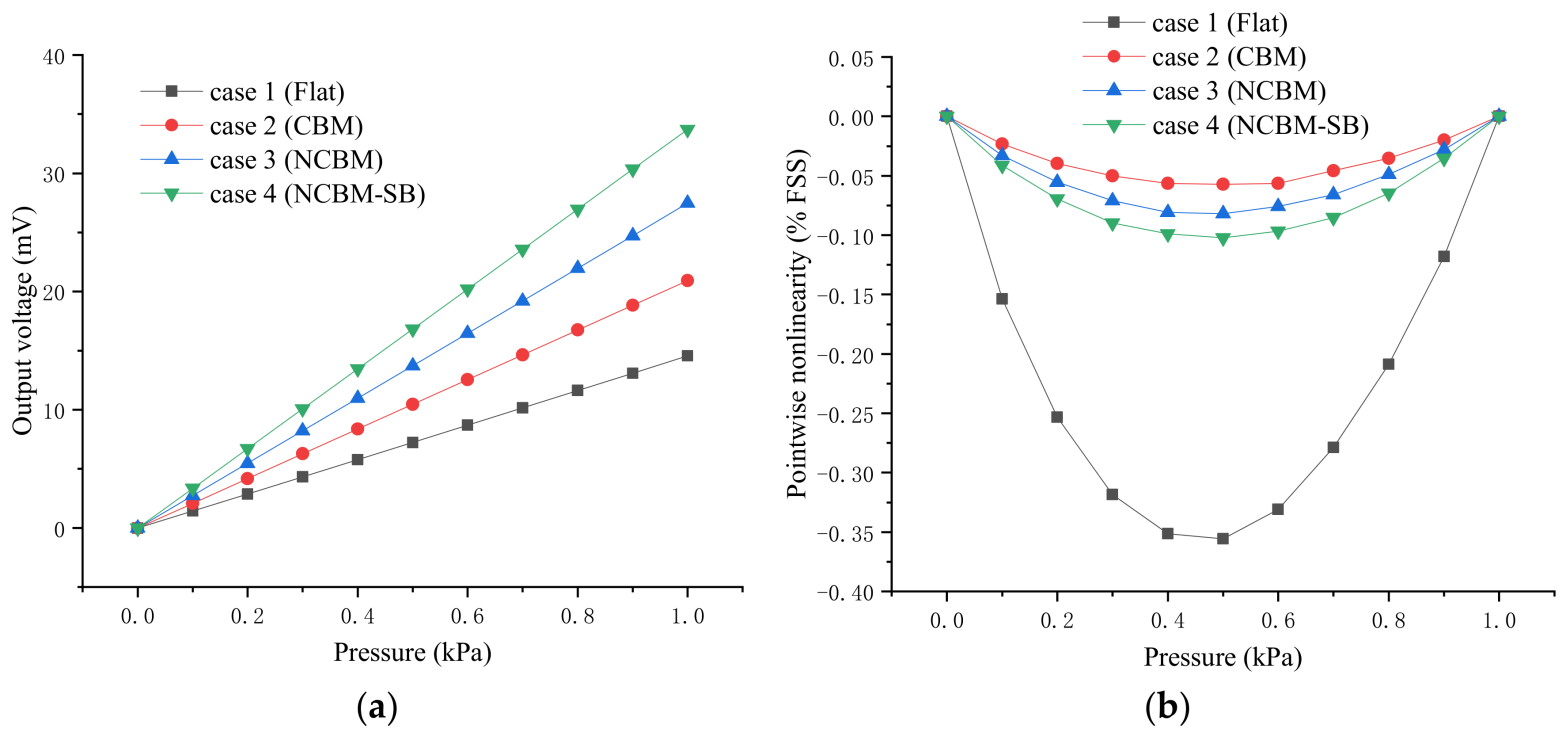
Comparison of the four structural configurations: (**a**) output voltage; (**b**) pointwise nonlinearity.

The bridge stress difference ${\Delta \sigma }_{bridge}$, output voltage, and nonlinear error of each configuration are compared in Table 2 and Figure 6. Case 4 exhibits the largest bridge stress difference and output voltage, followed sequentially by Cases 3, 2, and 1. Moreover, Cases 4, 3, and 2 exhibit comparable nonlinear errors, all of which are substantially lower than that of Case 1.

In summary, Case 4 provides the highest sensitivity while maintaining low nonlinear error. Its sensitivity is 61% higher than that of Case 2, indicating the best overall performance.

All four configurations satisfy the small-deflection criterion. Among them, Case 4 provides the largest bridge stress difference and output voltage while maintaining low nonlinear error. These results demonstrate that the proposed narrow cross-beam membrane–short beam structure effectively intensifies stress concentration and improves sensor sensitivity while maintaining favorable output linearity. Accordingly, Case 4 was selected as the baseline model for subsequent structural-parameter optimization.

## 4. Dimensional Optimization

According to the analytical equations for a conventional flat diaphragm, the mechanical bridge stress difference and maximum deflection are power functions of the structural dimensions. The functional relationships for the NCBM-SB structure may therefore be similar to those of a conventional flat diaphragm and can be approximated as follows [14,27]:

(12) $$∆\sigma_{bridge}=K_{1}pL^{n_{1}}H^{n_{2}}k^{n_{3}}a^{n_{4}}b^{n_{5}}\beta^{n_{6}}l^{n_{7}}$$

(13) $$\omega =K_{2}pE^{-1}L^{m_{1}}H^{m_{2}}k^{m_{3}}a^{m_{4}}b^{m_{5}}\beta^{m_{6}}l^{m_{7}}$$

**Figure 7:**
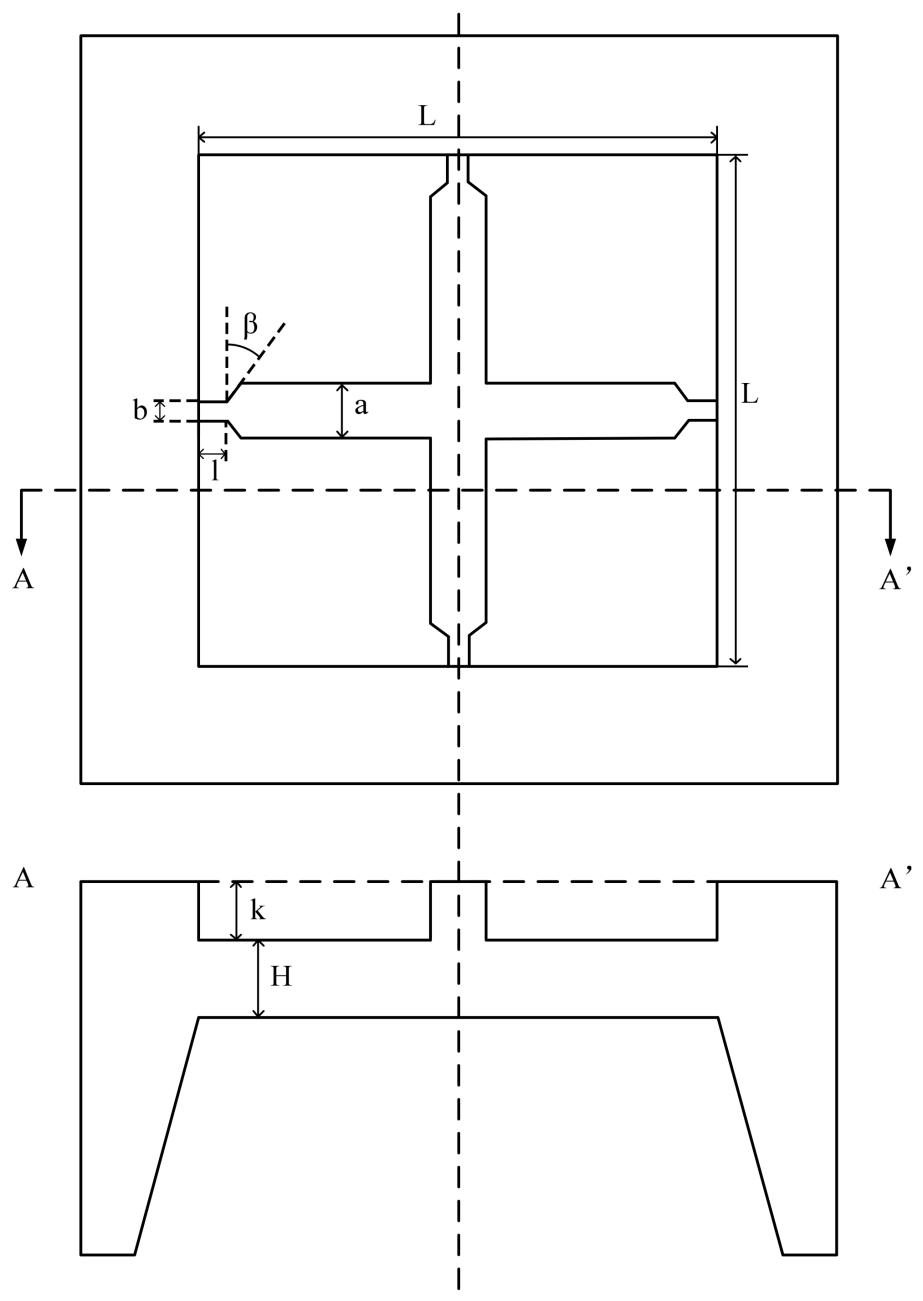
Top and A–A′ cross-sectional views defining the geometrical parameters; dielectric layers are omitted for clarity.

Here, $∆\sigma_{bridge}$ and ω are the bridge stress difference and maximum deflection, respectively; H and k are the sensitive-diaphragm thickness and beam height, respectively; L, a, b, β, and l are the in-plane structural dimensions shown in Figure 7; $K_{1}$ and $K_{2}$ are coefficients to be determined; p is the applied pressure; and E is Young’s modulus. As defined in Figure 7, *L* denotes the side length of the square sensitive diaphragm; *H* denotes the sensitive-diaphragm thickness; *k* denotes the cross-beam height above the diaphragm surface; *a* denotes the cross-beam width before tapering; *b* denotes the short-beam width; *l* denotes the short-beam length; and *β* denotes the taper angle at the transition from width *a* to width *b*. The lower view is the A–A′ cross section indicated in the top view. Since silicon constitutes the primary load-bearing layer of the diaphragm and beam structure, E in the analytical expressions denotes the Young’s modulus of silicon and was set to 170 GPa. The materials and thicknesses of the SiO_2_ and Si_3_N_4_ layers remained fixed throughout the parametric simulations and therefore were not introduced as additional fitting variables. Nevertheless, their contributions to the stiffness, stress distribution, and deflection of the multilayer structure were captured by the finite-element results and are consequently incorporated into the fitted coefficients K_1_ and K_2_ and the resulting fitted relationships.

Based on Equations (12) and (13), each variable was analyzed individually while all other variables were temporarily held constant, allowing the corresponding coefficients to be determined sequentially [27]. For example, when analyzing the influence of diaphragm side length L on sensor performance, all other variables were fixed at their reference values and L was varied over a physically reasonable range. Equations (12) and (13) can therefore be rewritten as follows:

(14) $$∆\sigma_{bridge}(L)=K_{1L}L^{n_{1}}$$

(15) $$\omega (L)=K_{2L}L^{m_{1}}$$

**Figure 8:**
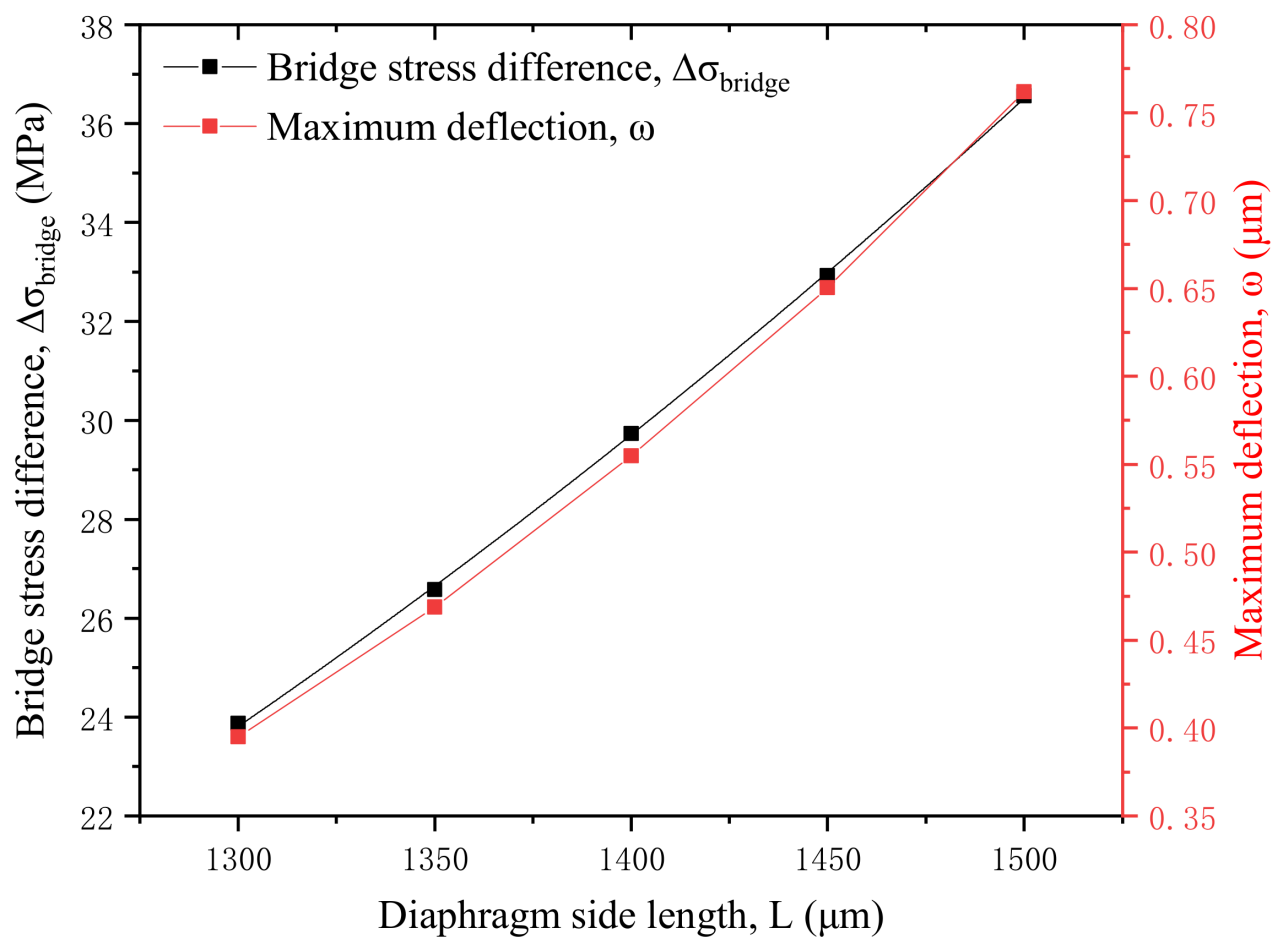
Bridge stress difference ${\Delta \sigma }_{bridge}$ and maximum central deflection ω as functions of diaphragm side length L.

As the diaphragm side length was varied, a series of deflection ω and bridge stress difference ${\Delta \sigma }_{bridge}$ values were obtained through finite-element simulations. Both the bridge stress difference at the resistor locations and the central diaphragm deflection increased with diaphragm side length. Numerical curve fitting was performed using these simulation results, as shown in Figure 8, yielding approximate power-law expressions for the deflection ω and bridge stress difference ${\Delta \sigma }_{bridge}$. The relationships between diaphragm side length L and structural performance are given by Equations (16) and (17).

(16) $$∆\sigma_{bridge}(L)=1.18\times 10^{-8}L^{2.99}$$

(17) $$\omega (L)=1.99\times 10^{-15}L^{4.59}$$

To evaluate the goodness of fit between the simulations and the preceding equations, Table 3 presents the fitted deflection and bridge stress difference functions together with their coefficients of determination ($R^{2}$) and residual sums of squares (RSS). The coefficients of determination for the bridge stress difference in Equation (16) ($R^{2}$) and the deflection in Equation (17) ($R^{2}$) are 0.99983 and 0.99998, respectively. The residual sums of squares for the bridge stress difference (RSS) and deflection (RSS) are 0.01687 and $1.72434\times 10^{-6}$, respectively. These results demonstrate excellent agreement between the power-law fitting equations and the simulations, confirming the validity of the mathematical model.

Similarly, functional expressions for the other variables can be obtained through simulations and power-law fitting. After all single-variable functions have been determined, they are substituted into Equations (12) and (13) to obtain the bridge stress difference and deflection equations applicable to the narrow cross-beam membrane–short beam (NCBM-SB) structure:

(18) $$∆\sigma_{bridge}=K_{1}pL^{2.99}H^{-1.09}k^{-0.704}a^{-0.0566}b^{-0.741}l^{-0.0477}$$

(19) $$\omega =K_{2}E^{-1}pL^{4.59}H^{-1.27}k^{-1.36}a^{-0.423}b^{-0.214}l^{0.0594}$$

**Figure 9:**
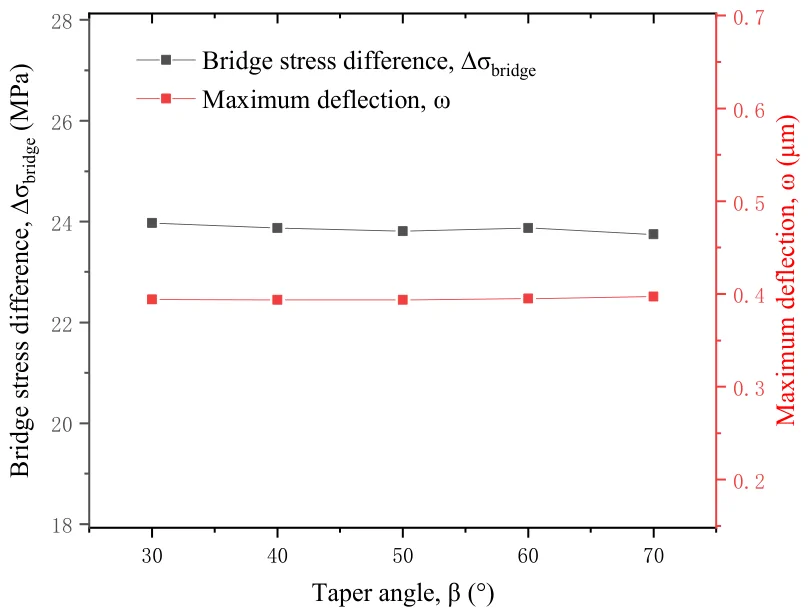
Bridge stress difference ${\Delta \sigma }_{bridge}$ and maximum deflection ω versus taper angle β.

It should be noted that parameter-sweep simulations of the taper angle β showed that different values of β produced only minor variations in the bridge stress difference and deflection, as illustrated in Figure 9. The influence of this variable on the sensor bridge stress difference and deflection can therefore be neglected, and it was excluded from the final expressions. Considering practical fabrication requirements, the taper angle was set to 60°.

Assume that the dimensional parameters in Equations (18) and (19) are L=1300 μm, H=5 μm, k=10 μm, a=60 μm, b=30 μm, l=35 μm, p=1 kPa, and E=170 GPa. The simulations yield a maximum deflection of approximately 0.40 μm and a maximum bridge stress difference of approximately 23.88 MPa. Substitution of these values into Equations (18) and (19) determines the coefficients $K_{1}$ and $K_{2}$. The final bridge stress difference and deflection equations for the narrow cross-beam membrane–short beam (NCBM-SB) structure can thus be expressed as follows:

(20) $$∆\sigma_{bridge}=0.00644pL^{2.99}H^{-1.09}k^{-0.704}a^{-0.0566}b^{-0.741}l^{-0.0477}$$

(21) $$\omega =0.00057E^{-1}pL^{4.59}H^{-1.27}k^{-1.36}a^{-0.423}b^{-0.214}l^{0.0594}$$

For a piezoresistive pressure sensor, sensitivity is proportional to the bridge stress difference [28]. Equations (20) and (21) indicate that the bridge stress difference is governed primarily by the diaphragm side length L, diaphragm thickness H, beam height k, and short beam width b, whereas the short beam length l and the cross-beam width before tapering a have relatively weak effects on sensitivity. The short beam length l likewise has only a minor influence on diaphragm deflection, whereas the cross-beam width before tapering a has a non-negligible influence on deflection.

Based on Equations (20) and (21), nonlinear optimization was performed using numerical analysis software:

(22) $$\left\{\begin{array}{c} max(∆\sigma_{bridge})\\ s.t\\ \omega \leq 0.2H\\ 1000\;\mu m\leq L\leq 1500\;\mu m\\ 4\;\mu m\leq H\leq 8\;\mu m\\ 6\;\mu m\leq k\leq 14\;\mu m\\ 40\;\mu m\leq a\leq 60\;\mu m\\ 30\;\mu m\leq b\leq 50\;\mu m\\ 35\;\mu m\leq l\leq 95\;\mu m \end{array}\right.$$

**Figure 10:**
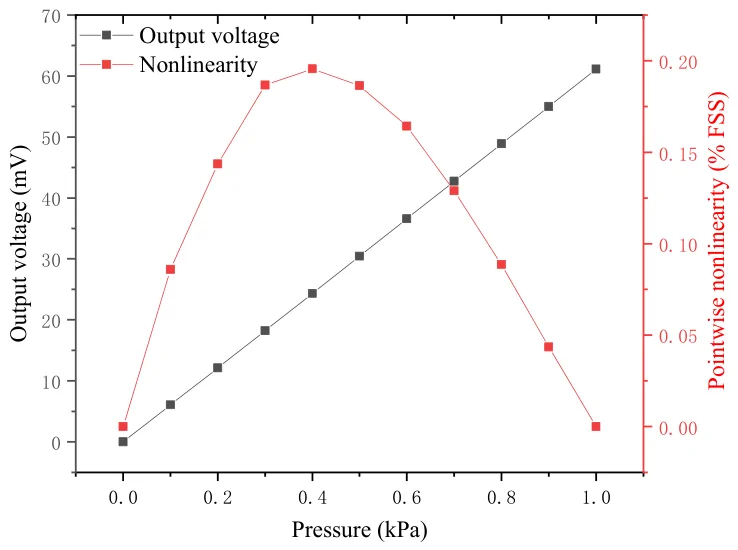
Output voltage and pointwise nonlinearity of the optimized NCBM-SB structure as functions of pressure.

The optimization results show that, when L=1500 μm, H=6.02 μm, k=6 μm, a=60 μm, b=30 μm, l=35 μm, p=1 kPa, and E=170 GPa, the bridge stress difference is approximately 42.80 MPa. The output voltage and pointwise nonlinearity of the sensor with these dimensions are shown in Figure 10. The corresponding sensitivity is 12.23 mV/V/kPa, and the maximum nonlinearity is 0.196% FSS.

It should be noted that several optimized geometrical parameters are located at the imposed constraint boundaries. This behavior is associated with the predominantly monotonic dependence of the bridge stress difference and maximum deflection on several key geometrical parameters within the validated design domain of the present fitting model. Therefore, under the imposed geometrical and deflection constraints, the optimizer naturally drives these parameters toward the corresponding boundaries as the objective function continues to improve in those directions. The resulting dimensions should thus be regarded as a constrained optimum within the validated design domain rather than a global optimum over the entire physically possible design space. Extending the optimization beyond the current domain would require further validation of the predictive accuracy of the fitting model in the enlarged design space.

## 5. Fabrication Feasibility Analysis

**Figure 11:**
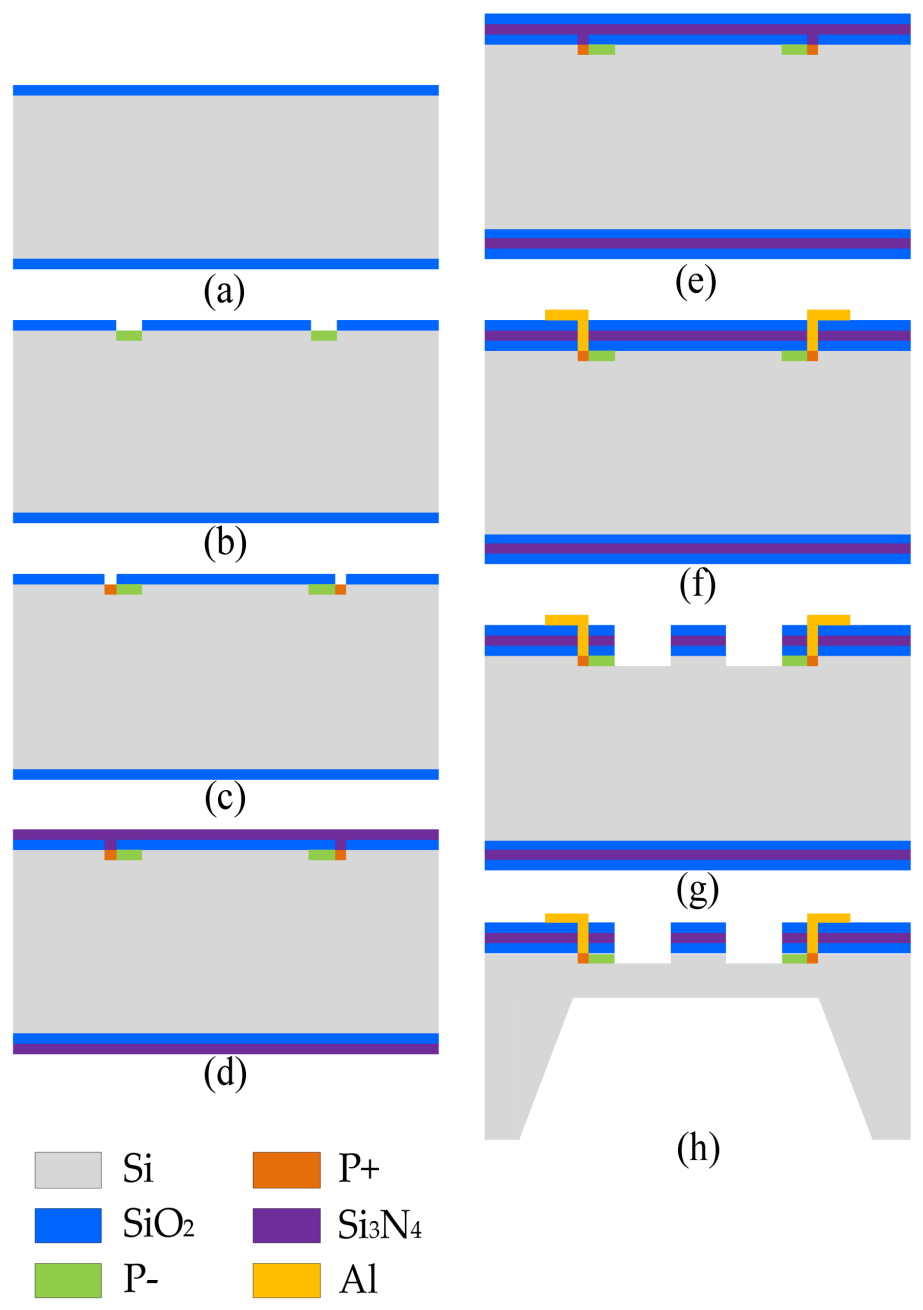
Principal fabrication process for the sensor chip. (**a**) Thermal oxidation; (**b**) P− piezoresistor formation; (**c**) P+ ohmic-contact formation; (**d**) Si_3_N_4_ passivation; (**e**) SiO_2_ insulation; (**f**) Al interconnection formation; (**g**) front-side NCBM-SB structure formation; (**h**) backside cavity etching and removal of the protective coating.

To assess the fabrication feasibility of the proposed narrow cross-beam membrane–short beam structure, a process flow based on conventional MEMS bulk-silicon micromachining was designed. A standard double-side-polished (100) silicon wafer with a thickness of 400 μm was used as the substrate. Seven photolithographic masks were employed to define the piezoresistors, sensitive structure, metal contacts, and backside cavity, and boron implantation was used to form the P-type piezoresistors. The principal fabrication steps are shown in Figure 11.

(1) The silicon wafer is thermally oxidized to form thin SiO_2_ layers on both surfaces. These oxide layers serve as masks during ion implantation (Figure 11a).
(2) After thermal oxidation, P−-region photolithography and boron-ion implantation are performed to form the P-type piezoresistors on the front surface of the silicon wafer (Figure 11b).
(3) P+-region photolithography and higher-dose boron-ion implantation are then performed to form ohmic contacts between the piezoresistors and Al (Figure 11c).
(4) A Si_3_N_4_ passivation layer is deposited by low-pressure chemical vapor deposition (LPCVD) to protect the piezoresistors (Figure 11d).
(5) An SiO_2_ film is subsequently deposited by LPCVD to provide electrical insulation (Figure 11e).
(6) A thin Al layer is deposited and patterned on the top surface of the sensor chip using a reactive ion etching (RIE) process to provide electrical interconnections between the piezoresistors and the Wheatstone bridge (Figure 11f).
(7) The narrow cross-beam membrane–short beam structure is then formed on the front surface of the silicon wafer by RIE (Figure 11g).
(8) Before backside wet etching, ProTEK B3 is spin-coated on the front surface of the wafer to protect the front-side structures from the tetramethylammonium hydroxide (TMAH) solution [29]. With the front surface protected, the backside of the silicon wafer is wet etched using TMAH [30] to form a deep cavity. After backside etching, the ProTEK B3 protective coating is removed (Figure 11h).

## 6. Performance Comparison and Discussion

To further evaluate the overall performance of the proposed sensor, its key performance metrics were compared with those of representative piezoresistive micropressure sensors reported in previous studies. The optimized NCBM-SB structure achieves a sensitivity of 12.23 mV/V/kPa and a maximum nonlinearity of 0.196% FSS over a pressure range of 0–1 kPa. Its sensitivity is higher than that of most of the reported structures and is lower only than those reported in [8,9]. Meanwhile, the nonlinearity of 0.196% FSS is also relatively low among the reported values. These results indicate that the proposed structure achieves relatively high sensitivity without a pronounced deterioration in linearity, thereby providing a favorable balance between sensitivity and linearity.

As shown in Table 4, the sensors reported in [8,9] exhibit higher sensitivities than the present design; however, both were reported over a pressure range of 0–0.5 kPa, which is only half of the 0–1 kPa range evaluated in this work. The sensor in [8] achieves a higher sensitivity but exhibits a considerably larger nonlinearity. The sensor in [9] combines high sensitivity with low nonlinearity and therefore demonstrates excellent overall performance, although its measurement range is narrower. Rather than pursuing the highest sensitivity alone, the proposed NCBM-SB sensor maintains relatively high sensitivity and low nonlinearity while extending the measurement range to 0–1 kPa, thereby achieving a favorable trade-off among sensitivity, linearity, and measurement range.

The favorable performance is mainly attributed to the synergistic effects of the narrowed cross beams and short beam segments. The narrowed cross beams enhance stress concentration in the piezoresistive regions, while the short beams further tailor the local stiffness and deformation characteristics, thereby improving the electrical response without a pronounced increase in nonlinearity.

## 7. Conclusions

This study focused on improving sensitivity and reducing nonlinearity by proposing a novel MEMS piezoresistive pressure sensor structure based on a narrow cross-beam membrane–short beam (NCBM-SB). To evaluate the effectiveness of the proposed structure, it was compared with a flat diaphragm, a conventional cross-beam diaphragm, and a tapered cross-beam diaphragm. The proposed structure increased sensitivity by 61% relative to the conventional cross-beam structure. Finite-element simulations were also conducted over a pressure range of 0–1 kPa to characterize diaphragm deformation and the resulting stress difference in the piezoresistive regions. Curve fitting was then used to establish the relationships between the structural dimensions and the diaphragm deflection and bridge stress difference, thereby providing quantitative guidance for the design of the NCBM-SB structure. Nonlinear optimization was further performed to determine the constrained optimal geometry within the validated design domain. With the optimized dimensions, the sensor achieved a sensitivity of 12.23 mV/V/kPa and a nonlinearity of 0.196% FSS. Finally, a fabrication process based on bulk micromachining was designed for the sensor chip. Compared with structural solutions that rely on complex three-dimensional processing or specialized material systems, the proposed process is relatively mature and offers favorable repeatability and batch-fabrication capability. In comparison with representative piezoresistive micropressure sensors reported in previous studies, the proposed sensor demonstrates a favorable balance among sensitivity, nonlinearity, and measurement range. Nevertheless, the present multivariable power-law fitting model has certain limitations, and the obtained dimensions should be regarded as a constrained optimum within the validated design domain. Future work will further expand the finite-element dataset and investigate more flexible data-driven surrogate models, such as neural networks, to capture complex parameter–response relationships and further improve the robustness and applicability of the optimization.

## Figures and Tables

**Table 1:** Comparison of representative MEMS piezoresistive pressure sensors reported in previous studies.

Year/Ref.	Structure	Pressure Range	Sensitivity	Nonlinearity
2012 [4]	Planar square diaphragm with cross beams	0–5 kPa	1.55 mV/V/kPa	0.09% FSS
2016 [5]	Four-beam structure (Simulation)	0–5 kPa	5.10 mV/V/kPa	0.75% FSS
2017 [6]	Four-beam structure (Experimental)	0–5 kPa	4.65 mV/V/kPa	0.25% FSS
2013 [7]	Beam–diaphragm–single-island structure	0–0.5 kPa	11.098 mV/V/kPa	3.046% FS
2016 [8]	Beam–diaphragm–double-island structure	0–500 Pa	17.339 mV/V/kPa	2.556% FS
2015 [9]	Beam–diaphragm–four-island structure	0–500 Pa	17.795 mV/V/kPa	0.1405% FS
2014 [10]	Peninsula-structured diaphragm	0–5 kPa	3.68 mV/V/kPa	0.36% FSS
2026 [11]	Square diaphragm–four-narrow-beam–central-island structure	0–10 kPa	1.59 mV/V/kPa	−0.22% FS
2018 [12]	Grooves–cross-beam diaphragm	0–6.894 kPa	4.48 mV/V/kPa	0.21% FSS
2020 [13]	Locally thinned diaphragm	0–100 Pa	2.11 mV/V/kPa	—
2017 [14]	Cross-beam–peninsula composite diaphragm	0–5 kPa	5.14 mV/V/kPa	−0.28% FSS
2018 [15]	Four-petal diaphragm with short narrow beams and central boss	0–5 kPa	6.93 mV/V/kPa	0.23% FSS
2022 [16]	Central circular island–cross beam–four peninsula–short narrow beam structure	0–5 kPa	4.54 mV/V/kPa	0.11% FSO

Note: FSS, full-scale span; FSO, full-scale output; FS, full scale. Sensitivities reported in mV/kPa were normalized to mV/V/kPa using the corresponding excitation voltages reported in the original references.

**Table 2:** Comparison of the bridge stress difference ${\Delta \sigma }_{bridge}$, output voltage $V_{out}$, and nonlinear error of the four configurations.

Case	Membrane	${\Delta \sigma }_{bridge}$ (MPa)	$V_{out}$ (mV)	Nonlinearity (% FSS)
1	Flat	8.49	14.57	0.36
2	CBM	12.12	20.94	0.06
3	NCBM	15.91	27.46	0.08
4	NCBM-SB	19.53	33.72	0.10

**Table 3:** Fitted-curve parameters for the bridge stress difference and deflection.

Parameters	${\Delta \sigma }_{bridge}$	ω
Equation	$y=a\times L^{b}$	$y=a\times L^{b}$
a	$1.18\times 10^{-8}\pm 1.9\times 10^{-9}$	$1.99\times 10^{-15}\pm 1.8\times 10^{-16}$
b	2.99±0.02	4.59±0.01
RSS	0.0169	$1.72\times 10^{-6}$
R2	0.99983	0.99998

**Table 4:** Focused comparison of the proposed sensor with previously reported high-sensitivity piezoresistive micropressure sensors.

Sensor	Pressure Range	Sensitivity	Nonlinearity
Ref. [8]	0–500 Pa	17.339 mV/V/kPa	2.556% FS
Ref. [9]	0–500 Pa	17.795 mV/V/kPa	0.1405% FS
Proposed sensor	0–1 kPa	12.23 mV/V/kPa	0.196% FSS

## Data Availability

The original contributions presented in this study are included in the article. Further inquiries can be directed to the corresponding authors.

## References

[1] Han X., Huang M., Wu Z., Gao Y., Xia Y., Yang P., Fan S., Lu X., Yang X., Liang L. (2023). Advances in high-performance MEMS pressure sensors: Design, fabrication, and packaging. Microsyst. Nanoeng..

[2] Song P., Ma Z., Ma J., Yang L., Wei J., Zhao Y., Zhang M., Yang F., Wang X. (2020). Recent progress of miniature MEMS pressure sensors. Micromachines.

[3] Jena S., Gupta A. (2021). Review on pressure sensors: A perspective from mechanical to micro-electro-mechanical systems. Sens. Rev..

[4] Tian B., Zhao Y., Jiang Z., Hu B. (2012). The design and analysis of beam-membrane structure sensors for micro-pressure measurement. Rev. Sci. Instrum..

[5] Li C., Cordovilla F., Jagdheesh R., Ocaña J.L. (2017). Design and optimization of a novel structural MEMS piezoresistive pressure sensor. Microsyst. Technol..

[6] Li C., Cordovilla F., Ocaña J.L. (2018). Design optimization and fabrication of a novel structural piezoresistive pressure sensor for micro-pressure measurement. Solid-State Electron..

[7] Yu Z., Zhao Y., Sun L., Tian B., Jiang Z. (2013). Incorporation of beams into bossed diaphragm for a high sensitivity and overload micro pressure sensor. Rev. Sci. Instrum..

[8] Meng X., Zhao Y. (2016). The design and optimization of a highly sensitive and overload-resistant piezoresistive pressure sensor. Sensors.

[9] Yu Z., Zhao Y., Li L., Li C., Liu Y., Tian B. (2015). Realization of a micro pressure sensor with high sensitivity and overload by introducing beams and islands. Microsyst. Technol..

[10] Huang X., Zhang D. (2014). A high sensitivity and high linearity pressure sensor based on a peninsula-structured diaphragm for low-pressure ranges. Sens. Actuators A Phys..

[11] Yang Z., Tang Y., Tang F., Xie B., Ran X., Xie H. (2026). A high-sensitivity MEMS piezoresistive pressure sensor for intracranial pressure monitoring. Micromachines.

[12] Li C., Cordovilla F., Jagdheesh R., Ocaña J.L. (2018). Design optimization and fabrication of a novel structural SOI piezoresistive pressure sensor with high accuracy. Sensors.

[13] Kordrostami Z., Hassanli K., Akbarian A. (2020). MEMS piezoresistive pressure sensor with patterned thinning of diaphragm. Microelectron. Int..

[14] Tran A.V., Zhang X., Zhu B. (2018). The development of a new piezoresistive pressure sensor for low pressures. IEEE Trans. Ind. Electron..

[15] Tran A.V., Zhang X., Zhu B. (2018). Mechanical structural design of a piezoresistive pressure sensor for low-pressure measurement: A computational analysis by increases in the sensor sensitivity. Sensors.

[16] Gao C., Zhang D. (2022). The establishment and verification of the sensitivity model of the piezoresistive pressure sensor based on the new peninsula structure. J. Microelectromech. Syst..

[17] Liu Y., Jiang X., Yang H., Qin H., Wang W. (2023). Structural engineering in piezoresistive micropressure sensors: A focused review. Micromachines.

[18] Xu T., Zhao L., Jiang Z., Guo X., Ding J., Xiang W., Zhao Y. (2016). A high sensitive pressure sensor with the novel bossed diaphragm combined with peninsula-island structure. Sens. Actuators A Phys..

[19] Barlian A.A., Park W.T., Mallon J.R., Rastegar A.J., Pruitt B.L. (2009). Review: Semiconductor piezoresistance for microsystems. Proc. IEEE.

[20] Meng Q., Lu Y., Wang J., Chen D., Chen J. (2021). A piezoresistive pressure sensor with optimized positions and thickness of piezoresistors. Micromachines.

[21] Smith C.S., Seitz F., Turnbull D. (1958). Macroscopic symmetry and properties of crystals. Solid State Physics: Advances in Research and Applications.

[22] Li F., Yu R., Zhang D. (2023). An In-Situ Tester for Extracting Piezoresistive Coefficients. Micromachines.

[23] Bertagnoli G., Abbasi Gavarti M., Ferrara M. (2024). Ceramic Stress Sensor Based on Thick Film Piezo-Resistive Ink for Structural Applications. Sensors.

[24] Tian B., Zhao Y., Jiang Z. (2010). The novel structural design for pressure sensors. Sens. Rev..

[25] Deng S., Li F., Cai M., Jiang Y. (2024). Novel Flexible Pressure Sensor with Symmetrical Structure Based on 2-D MoS_2_ Material on Polydimethylsiloxane Substrate. Symmetry.

[26] Wu J., Zhao X., Liu Y., Wen D. (2020). The simulation, fabrication technology and characteristic research of micro-pressure sensor with isosceles trapezoidal beam-membrane. Mod. Phys. Lett. B.

[27] Ren X., Liu X., Su X., Jiang X. (2022). Design and optimization of a pressure sensor based on serpentine-shaped graphene piezoresistors for measuring low pressure. Sensors.

[28] Guan T., Yang F., Wang W., Huang X., Jiang B., He J., Zhang L., Fu F., Li D., Li R. A novel 0–3 kPa piezoresistive pressure sensor based on a shuriken-structured diaphragm. Proceedings of the 2016 IEEE 29th International Conference on Micro Electro Mechanical Systems (MEMS).

[29] Zhang X., Yamaner F.Y., Oralkan Ö. (2017). Fabrication of Vacuum-Sealed Capacitive Micromachined Ultrasonic Transducers With Through-Glass-Via Interconnects Using Anodic Bonding. J. Microelectromech. Syst..

[30] Tabata O., Asahi R., Funabashi H., Shimaoka K., Sugiyama S. (1992). Anisotropic etching of silicon in TMAH solutions. Sens. Actuators A Phys..

